# A general strategy for expanding polymerase function by droplet microfluidics

**DOI:** 10.1038/ncomms11235

**Published:** 2016-04-05

**Authors:** Andrew C. Larsen, Matthew R. Dunn, Andrew Hatch, Sujay P. Sau, Cody Youngbull, John C. Chaput

**Affiliations:** 1 The Biodesign Institute, Arizona State University, Tempe, Arizona 85287-5301, USA; 2 School of Life Sciences, Arizona State University, Tempe, Arizona 85287-5301, USA; 3 School of Earth and Space Exploration, Arizona State University, Tempe, Arizona 85287-5301, USA; 4 Department of Chemistry and Biochemistry, Arizona State University, Tempe, Arizona 85287-5301, USA; 5 Department of Pharmaceutical Sciences, University of California, 147 Bison Modular, Building 515, Irvine, California 92697

## Abstract

Polymerases that synthesize artificial genetic polymers hold great promise for advancing future applications in synthetic biology. However, engineering natural polymerases to replicate unnatural genetic polymers is a challenging problem. Here we present droplet-based optical polymerase sorting (DrOPS) as a general strategy for expanding polymerase function that employs an optical sensor to monitor polymerase activity inside the microenvironment of a uniform synthetic compartment generated by microfluidics. We validated this approach by performing a complete cycle of encapsulation, sorting and recovery on a doped library and observed an enrichment of ∼1,200-fold for a model engineered polymerase. We then applied our method to evolve a manganese-independent α-L-threofuranosyl nucleic acid (TNA) polymerase that functions with >99% template-copying fidelity. Based on our findings, we suggest that DrOPS is a versatile tool that could be used to evolve any polymerase function, where optical detection can be achieved by Watson–Crick base pairing.

## Abstract (web summary)

Droplet-based optical polymerase sorting employs a fluorescent sensor to monitor polymerase activity inside the microenvironment of uniform water-in-oil emulsions. Here, the authors use this technique to select and isolate single cells for evolution of an unnatural nucleic acid polymerase.

## Graphical abstract

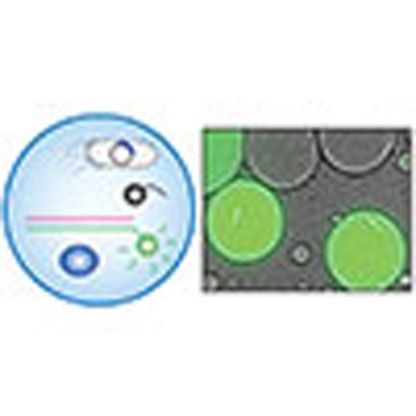

Recent advances in polymerase engineering have made it possible to synthesize nucleic acid polymers with a wide range of chemical modifications, including xeno-nucleic acid polymers (XNAs) with backbone structures that are not found in nature^1^,^2^,^3^. While this technological advance has generated significant interest in XNA as a synthetic polymer for future applications in molecular medicine, nanotechnology and materials science^4^,^5^,^6^,^7^, the current generation of XNA polymerases function with markedly lower activity than their natural counterparts^8^,^9^. The prospect of developing synthetic polymerases with improved activity and more diverse functions has driven a desire to apply molecular evolution as a strategy for altering the catalytic properties of natural polymerases^10^,^11^. Compartmentalized self-replication (CSR) and compartmentalized self-tagging (CST) are examples of technologies that have been developed to evolve polymerases with expanded substrate specificity^1^,^12^. However, these methods use the parent plasmid as template for the primer-extension reaction, which limits the range of polymerase functions to enzymes that promote DNA-templated synthesis.

Evolving enzymes with new or improved function requires iterative rounds of *in vitro* selection and amplification^13^. The outcome of a selection depends on the number of variants that can be screened and the quality of the separation technique used to partition functional members away from the non-functional pool. The miniaturization of directed evolution experiments into artificial compartments with cell-like dimensions provides access to larger enzyme libraries by reducing sample volumes to the picolitre-scale^14^,^15^. The simplest approach to water-in-oil (w/o) droplet formation involves the bulk mixing of aqueous and organic phases with vigorous stirring, but this method produces polydisperse droplets with large volumetric differences^14^,^15^. Given the cubic dependence of volume on diameter, polydisperse droplets cannot be partitioned by optical sorting due to massive differences in enzyme–substrate concentration^16^.

To overcome this problem, microfluidic devices have been developed that can generate monodisperse populations of w/o droplets by manipulating fluids at the microscale^17^,^18^. While this approach has been used to change the specificity of several natural enzymes^19^,^20^,^21^, this technique has not yet been applied to problems in polymerase engineering due to the challenges of generating a fluorescent signal with a signal-to-noise ratio (SNR) that is high enough to distinguish droplets containing functional polymerases from those that are empty or contain non-functional enzymes. Here we describe a microfluidics-based polymerase engineering strategy that combines droplet microfluidics with optical cell sorting. Using droplet-based optical polymerase sorting (DrOPS), a library of polymerase variants is expressed in *Escherichia coli* and single cells are encapsulated in microfluidic droplets containing a fluorescent substrate that is responsive to polymerase activity. On lysis, the polymerase is released into the droplet and challenged to extend a primer–template complex with XNA. Polymerases that successfully copy a template strand into full-length product produce a fluorescent signal by disrupting a donor–quencher pair. Although we originally developed the DrOPS method to evolve a manganese-independent TNA polymerase, the generality of this technique suggests that it could be used to evolve any polymerase function where optical detection can be achieved by Watson–Crick base pairing.

## Results

### Fluorescence-based PAA

**Figure 1:**
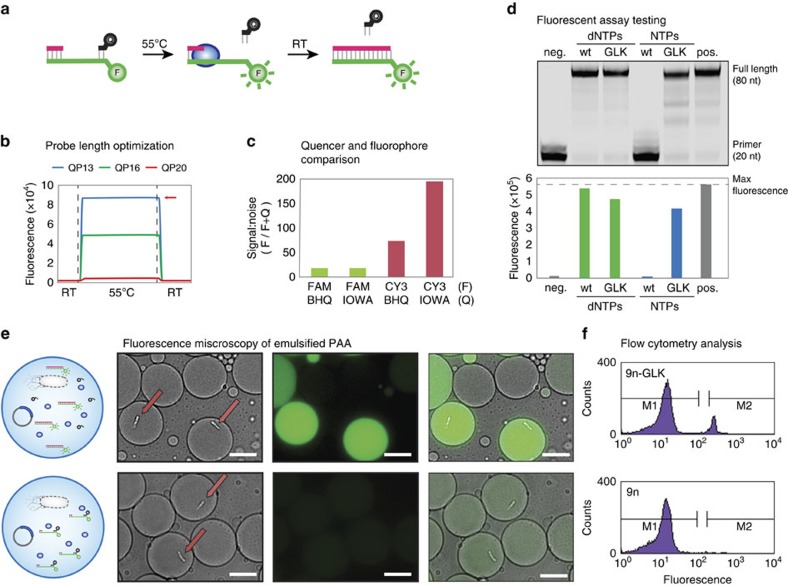
Droplet-based optical polymerase sorting. (**a**) We have developed a fluorescent reporter system that produces an optical signal when a primer–template complex is extended to full-length product. The reporter consists of a primer–template complex (pink and green) containing a downstream fluorophore that is quenched when a DNA-quencher (black) anneals to the unextended region. (**b**) The assay was designed with a metastable probe to allow dissociation at elevated temperatures, where thermophilic polymerases function with optimal activity. Red arrow marks the maximium fluorescence observed in the absence of the quencher probe. (**c**) Flourophore (F)/quencher (Q) pairs were screened to identify a dye pair with the maximum signal-to-noise ratio. (**d**) Primer-extension analysis by denaturing PAGE (top) and fluorescence (bottom) for 9n and 9n-GLK polymerases using dNTP and NTP substrates. Negative control: no NTPs. Positive control: dNTPs or no DNA-quencher probe. (**e**) Single-emulsion droplets containing a functional 9n-GLK polymerase that extends a primer–template complex with RNA (top) and non-functional (bottom) wild-type 9n polymerase. The panel shows a cartoon depiction of the droplet, a bright-field micrograph of encapsulated *E. coli* (arrow), a fluorescence micrograph of the same field of view and an overlay of the two images. Scale bars, 10 μm. (**f**) Flow cytometry analysis of 9n and 9n-GLK polymerases following NTP extension in water-in-oil-in-water (w/o/w) droplets.

Molecular beacons previously developed to monitor polymerase function suffer from a low SNR that precludes their use in w/o droplets^22^,^23^. We therefore set out to design a polymerase activity assay (PAA) that would produce a strong optical signal when a primer–template complex is extended to full-length product, but remain dim when the primer goes unextended (Fig. 1a). With this goal in mind, a DNA-quencher probe was designed to dissociate from the primer–template complex at elevated temperatures where thermophilic polymerases function with optimal activity and re-anneal at room temperature when the sample is assayed for function (Fig. 1b). By coupling polymerase activity to fluorescence, genes encoding functional polymerases are identified by the optical signal of their droplet, while variants that fail to extend the primer remain dim and are removed from the pool during cell sorting.

Recent advances in the chemistry of dark quenchers caused us to speculate that a donor–quencher pair could be identified with improved spectral properties^24^. By surveying a small number of fluorescent dyes, we found that Cy3 produces an optical signal that is 200-fold higher than its quenched state with Iowa Black FQ (Fig. 1c), which is substantially higher than previous donor–quencher pairs developed to monitor polymerase activity^22^.

To test the Cy3-Iowa Black FQ donor–quencher pair in a PAA, the RNA synthesis activity of an engineered DNA polymerase was compared with its wild-type (wt) DNA polymerase counterpart. For this experiment, we used 9n-GLK, which is an engineered version of a DNA polymerase isolated from *Thermococcus* sp. 9° N that carries the mutations Y409G, A485L and E664K (ref. 25). Exonuclease-deficient versions of 9n-GLK and wt 9n were challenged to extend a DNA primer–template complex with deoxyribonucleoside triphosphates (dNTP) and ribonucleoside triphosphates (NTP). Analysis of the primer-extension reactions by denaturing PAGE and fluorescence confirmed that full-length product is obtained in all cases except when the wt polymerase is incubated with NTPs (Fig. 1d). This result is consistent with the strong steric gate activity of natural DNA polymerases^26^. More importantly, however, the strong concordance observed between the PAGE and fluorescence-analysed data (Fig. 1d) demonstrates that the Cy3-Iowa Black FQ donor–quencher produces an optical signal suitable for monitoring polymerases activity in a bulk aqueous environment.

### Miniaturizing the PAA

Next, we sought to miniaturize the PAA by encapsulating the primer–template complex in uniform w/o droplets. We began by making w/o droplets in a flow-focusing, fluorocarbon-coated microfluidic device (Supplementary Fig. 1). In this system, droplet formation occurs at the flow-focusing junction where the aqueous phase meets a fluorous oil carrier phase. The droplets are stabilized by surfactants in the oil that prevent coalescence at elevated temperatures (as high as 90 °C) and allow for long-term storage at room temperature.

To demonstrate that the PAA functions within the environment of a w/o droplet, strains of *E. coli* expressing the wt and 9n-GLK mutant polymerases were encapsulated with the reagents needed for RNA synthesis on a DNA primer–template complex. Droplets were formed following a Poisson distribution (*μ*=0.1) to ensure that 99% of the occupied droplets contain at most a single *E. coli* cell. This prediction was empirically validated using cells expressing the green fluorescence protein (GFP; Supplementary Fig. 2). Once formed, the droplets were heated to promote *E. coli* lysis and incubated for 3 h at 55 °C to facilitate primer extension. Fluorescence and bright-field images were taken to assess polymerase activity in a population of w/o droplets. As shown in Fig. 1e, droplets containing the 9n-GLK *E. coli* strain produce a highly fluorescent signal due to the strong RNA synthesis activity of 9n-GLK, while empty droplets or droplets that contain the wild-type 9n *E. coli* strain remain dim. Taken together, these images demonstrate that the PAA functions with high activity in uniform w/o compartments, which is a necessary criterion for developing a microfluidics-based method for polymerase evolution.

### Formation of double-emulsion droplets

While w/o droplets provide a physical barrier for maintaining the genotype-phenotype linkage of functional enzymes, the organic carrier phase poses an obstacle for isolating fluorescent droplets using a commercial fluorescence-activated cell sorter (FACS). This problem can be overcome by performing a second compartmentalization step in which w/o droplets are emulsified in water-in-oil-in-water (w/o/w) double-emulsion droplets that have an aqueous carrier phase^27^. We therefore prepared a set of double-emulsion compartments using a hydrophilic microfluidics device that combines the w/o droplets with an aqueous carrier phase at the flow-focusing junction (Supplementary Fig. 1). Two populations of single-emulsion droplets containing either 9n-GLK or wt 9n DNA polymerase were converted to w/o/w droplets and analysed by flow cytometry (Fig. 1f). The population generated with *E. coli* cells expressing the wt 9n polymerase display uniformly low fluorescence, while droplets generated with *E. coli* cells expressing 9n-GLK have a bimodal distribution with low and high fluorescence. The fraction of highly fluorescent droplets correlates with the expected bacterial occupancy of ∼10% as predicted by statistical analysis and the GFP encapsulation assay (Supplementary Fig. 2). Moreover, the difference in average fluorescence intensity between the two populations is >10-fold, which is sufficient to separate the two populations by FACS.

### Enrichment efficiency

**Figure 2:**
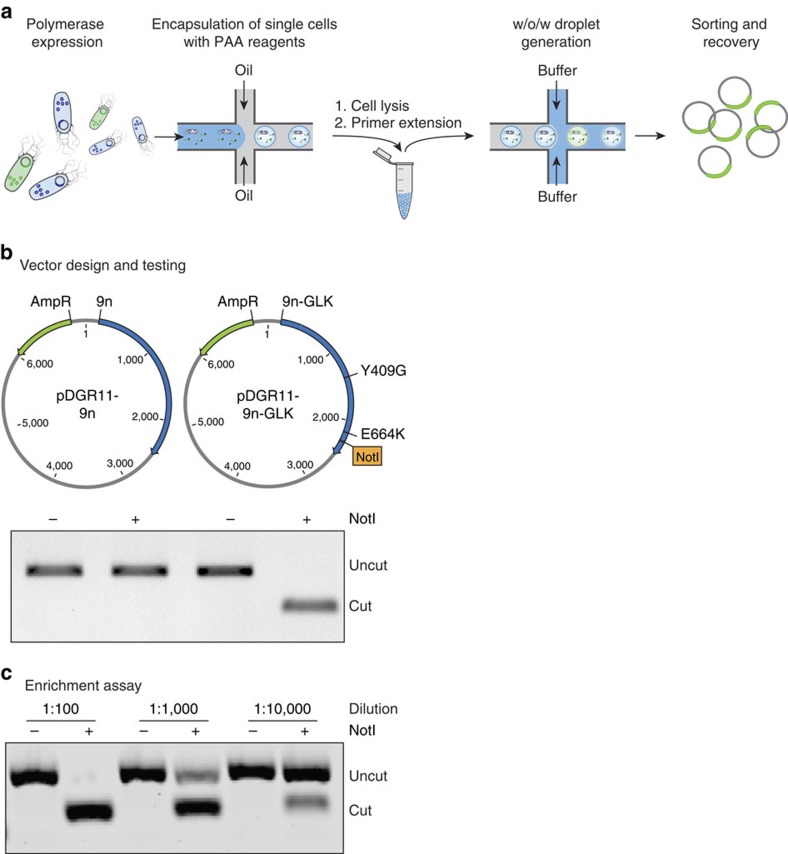
Model selection of an engineered polymerase with RNA synthesis activity. (**a**) Overview of the microfluidic polymerase enrichment strategy. A pool of polymerase genes containing functional (green) and non-functional (blue) members are expressed in *E. coli* and encapsulated in w/o droplets generated in a microfluidics device. Polymerases are liberated from their bacteria by heat lysis and incubated at 55 °C to allow for primer extension. Using a second microfluidics device, droplets are emulsified into a bulk aqueous phase to generate water-in-oil-in-water compartments (w/o/w). Fluorescent w/o/ws are FACS sorted and the vectors encoding functional polymerases are recovered. (**b**) Vector design. The 9n-GLK vector was engineered to contain a unique NotI restriction site. Control digestion showing that NotI only cuts PCR-amplified DNA from the 9n-GLK vector. (**c**) Following a complete cycle of selection and amplification (see Supplementary Fig. 1) PCR-amplified DNA was digested with NotI to measure the enrichment of 9n-GLK from libraries that were doped at levels of 1:100, 1:1,000 and 1:10,000 (9n-GLK to 9n). NotI digestion of the PCR-amplified DNA reveals an enrichment of ∼1,200-fold per round of microfluidics selection.

To test the ability of the PAA to support a complete round of *in vitro* selection (Fig. 2a, Supplementary Fig. 3), we performed a mock selection to measure the amount of enrichment that occurs per round of selection using the DrOPS method. *E. coli* cells expressing the 9n wt polymerase were combined with 1/100th, 1/1,000th and 1/10,000th of one equivalent of *E. coli* cells expressing 9n-GLK as a positive control for RNA synthesis activity. The 9n-GLK plasmids were engineered to contain a unique NotI restriction site to distinguish 9n-GLK from wt 9n in a restriction enzyme digestion (Fig. 2b). Accordingly, the three populations of *E. coli* were encapsulated in w/o droplets at an occupancy level of ∼10%, which ensured that 99% of the occupied droplets contained no more than one *E. coli* per compartment. Following cell lysis and primer extension, the samples were passed through a second microfluidics device to generate three populations of w/o/w droplets that were each sorted by FACS (Supplementary Fig. 1). Plasmid DNA recovered from the different populations was amplified by PCR and digested with NotI restriction enzyme. Comparison of the digested DNA before and after sorting revealed an enrichment of ∼1,200-fold of 9n-GLK (Fig. 2b,c), which is consistent with previous literature results where model libraries have been sorted in w/o/w double-emulsion droplets^27^.

### Evolving a manganese-independent TNA polymerase

**Figure 3:**
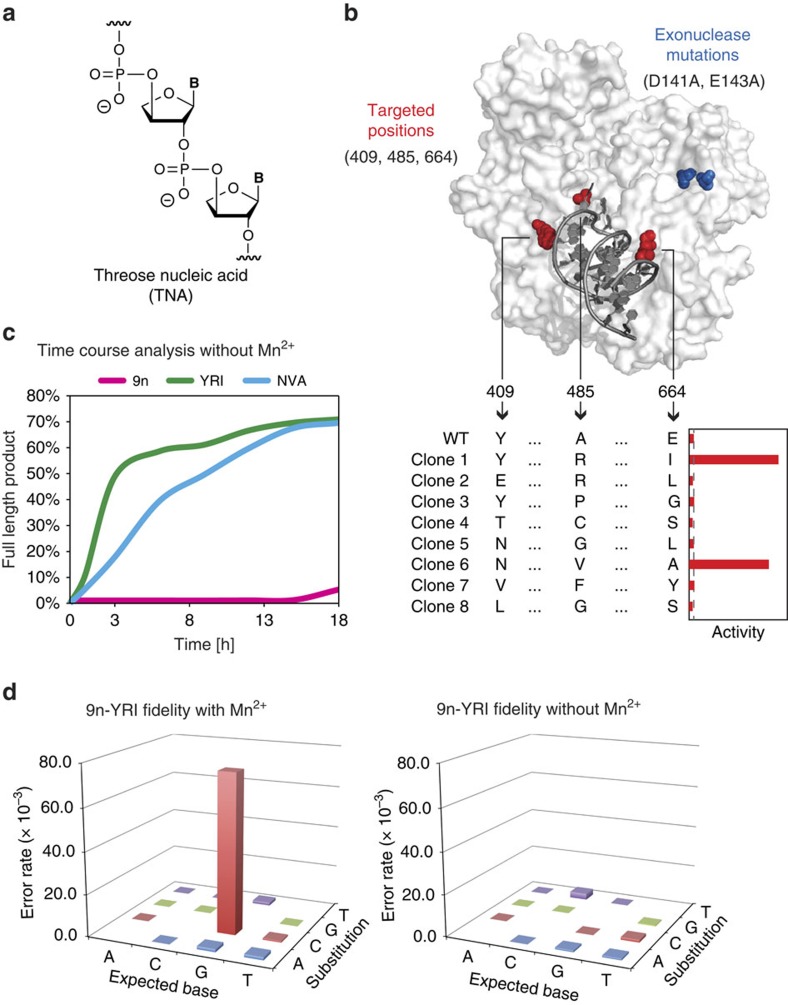
Selection of a Mn^2+^-independent TNA polymerase from a focused library. (**a**) Constitutional structure for the linearized backbone of threose nucleic acid (TNA). (**b**) Positions 409, 485 and 664 mapped onto the structure of 9n DNA polymerase (PDB: 4K8X). Polymerases isolated after one round of selection were analysed for TNA synthesis activity in the absence of Mn^2+^. Activity is defined as the amount of full-length product generated in 18 h. Basal activity of wild-type 9n polymerase (dashed grey line). (**c**) Time course of TNA synthesis for 9n-YRI and 9n-NVA polymerases compared with wild-type 9n. (**d**) Fidelity analysis of 9n-YRI polymerase in the presence and absence of manganese ions yields a mutational profile of 8 errors per 100 bases and 2 errors per 1,000 bases, respectively.

To demonstrate the DrOPS technology in a practical application, we sought to evolve a polymerase that could synthesize an artificial genetic polymer with a backbone structure unrelated to natural DNA and RNA. For this experiment, we chose α-L-threofuranosyl nucleic acid (TNA)—an unnatural genetic polymer composed of repeating units of α-L-threofuranosyl sugars linked by 2′,3′-phosphodiester bonds (Fig. 3a)^28^. TNA is an attractive candidate for therapeutic and diagnostic applications due to its stability against nuclease degradation and ability to undergo Darwinian evolution^3^. However, the current generation of TNA polymerases suffers from low fidelity due to a propensity for G–G mispairing in the enzyme active site^29^.

We hypothesized that the low fidelity of TNA synthesis was due to the presence of manganese ions (Mn^2+^), which are used to relax the substrate specificity of natural polymerases^30^. We therefore designed an *in vitro* selection strategy to evolve a Mn^2+^-independent TNA polymerase in hopes of generating an enzyme that functions with higher fidelity. A polymerase library was constructed in which positions 409, 485 and 664 in the 9n DNA polymerase scaffold were fully saturated with all possible amino acid mutations. These positions were chosen based on their known propensity to alter the substrate specificity of natural polymerases^31^. The 8,000 member library was assembled from commercial gene blocks (Supplementary Fig. 4), cloned into *E. coli* and sequence verified. Because the sequencing results revealed a number of random mutations in the gene-coding region, including unwanted stop codons, a single round of selection was performed under standard DNA synthesis conditions to increase the proportion of active clones. SDS–PAGE analysis of randomly selected clones before and after active polymerase enrichment revealed a dramatic increase in the number of full-length enzymes, indicating that neutral selection removed the truncated non-functional polymerases from the pool (Supplementary Fig. 5).

Next, the plasmid library was taken through a complete round of *in vitro* selection and amplification (Supplementary Fig. 3). Following w/o droplet formation and *E. coli* lysis, the polymerases were challenged to extend a DNA primer–template complex with chemically synthesized TNA triphosphates (tNTPs) in manganese-deficient reaction buffer for 18 h at 55 °C (refs 32, 33). The w/o droplets were converted to double emulsions and sorted by FACS. Plasmid DNA was extracted, transformed into a new population of *E. coli* and library members were cloned and sequenced.

### Characterizing selected TNA polymerases

Eight polymerase variants were chosen for functional analysis (Fig. 3b). Each polymerase was purified by affinity chromatography, quantified and assayed for the ability to extend a DNA primer–template complex with chemically synthesized tNTPs. Control experiments performed in the presence and absence of dNTP substrate confirmed that each polymerase was functional and free of cellular contaminants that could lead to a false positive result in the PAA (Supplementary Fig. 6). Of the eight polymerases tested, two variants showed a significant propensity for TNA synthesis in the absence of manganese ions (Fig. 3b).

Clone 1 (9n-YRI) carries the mutations A485R and E664I and retains the wt tyrosine residue (Y) at position 409. Clone 6 (9n-NVA) carries the mutations Y409N, A485V and E664A, as well as two additional point mutations (D432G and V636A). A time course analysis comparing 9n-YRI and 9n-NVA with wt 9n (Fig. 3c) indicates that both engineered polymerases function as strong Mn^2+^-independent TNA polymerases, generating ∼50% full-length product in 3 h and 9 h, respectively. By contrast, wt 9n shows very little full-length product after 18 h of incubation under identical conditions (Fig. 3c, Supplementary Fig. 6), indicating that the selected mutations enable 9n DNA polymerase to synthesize TNA in the absence of manganese ions.

### TNA replication fidelity

The strong TNA synthesis efficiency of 9n-YRI provided an opportunity to compare the effect of manganese ions on the fidelity of TNA synthesis. We therefore measured the fidelity of TNA synthesis by sequencing >2,000 nucleotide positions isolated from the complementary DNA (cDNA) product generated after a complete cycle of TNA replication (DNA→TNA→DNA) (Supplementary Fig. 7). Unlike kinetic fidelity assays which examine a single-nucleotide insertion event^34^, DNA sequencing provides a more complete view of the replication cycle by identifying insertions, deletions and mutations that occur when genetic information is converted from DNA into TNA and then in a separate reaction from TNA back into DNA^1^.

A series of controls was used to ensure that the sequencing data reflected the accuracy of TNA ‘transcription' and ‘reverse transcription' in the primer-extension reactions. The first control was a PCR assay that tested for DNA contaminants in the TNA product isolated by PAGE purification (Supplementary Fig. 8). In no cases did we observe a PCR product that amplified with the same number of cycles as the cDNA strand isolated from the reverse transcription of a TNA template into DNA. The second control involved checking the sequencing product to ensure that a T to A mutation occurred in the primer-binding site. Our TNA synthesis reaction was performed with a primer that contained a single-nucleotide mismatch that would lead to a T to A mutation when the TNA strand was reverse transcribed into DNA but lacking in sequences that were amplified from DNA contaminants^3^ (Supplementary Table 2).

Analysis of the sequencing results indicates that a TNA replication cycle performed with 9n-YRI as the TNA polymerase and SuperScript II as the reverse transcriptase produces ∼2 mistakes out of 1,000 nucleotide incorporations when manganese ions are absent from the TNA synthesis reaction. By contrast, the mutation rate is ∼50-fold higher when the same reaction was performed in the presence of manganese ions (Fig. 3d). This striking result confirms the hypothesis that manganese ions lower the fidelity of TNA synthesis and provide a viable strategy for faithful TNA synthesis under conditions that more closely approximate natural DNA synthesis. In this regard, 9n-YRI and 9n-NVA, represent the first demonstration of TNA polymerases that functions in the absence Mn^2+^ (Fig. 3c and Supplementary Fig. 6b).

## Discussion

Synthetic genetics aims to develop artificial genetic polymers that can replicate *in vitro* and eventually in model cellular organisms^4^. Achieving this ambitious goal will require major advances in chemical synthesis and polymerase engineering, as both fields of science are needed to develop the tools necessary for copying information back and forth between DNA and XNA and eventually between XNA polymers themselves. Recognizing that some of the most interesting XNAs can only be obtained by chemical synthesis^7^, researchers are facing a pressing need for new synthetic protocols that can be used to generate XNA monomers on the gram scale. Coupled with this effort is the equally challenging demand for new XNA polymerases that can synthesize kilobases of information with no mistakes. While this later goal may seem modest in comparison to natural polymerases, which can faithfully copy a megabase of DNA, the applications envisioned for XNA are less demanding than the biological requirements imposed by cellular organisms^35^.

In the current study, we present DrOPS as a new strategy for engineering polymerases with non-natural functions. Our method relies on single and double-emulsion droplets that are produced using commercially available microfluidic chips and reagents. The two-chip design simplifies the procedure for generating monodisperse droplets and provides flexibility for controlling such parameters as droplet size and oil-layer thickness^27^. With this system, droplets can be produced and screened in a matter of hours, which allows a round of selection to take place in 3–4 days. For example, we screened a library of 36 million double-emulsion droplets in 2 h (at 5 kHz) by fluorescence-activated cell sorting. Based on this rate of sorting, we suggest that it should be possible to screen >10^8^ droplets per day, which may be necessary for some polymerase functions that require greater library diversity.

The DrOPS method has several advantages over existing polymerase engineering technologies. Relative to screening procedures that assay variants in microlitre-scale reactions, miniaturization of the PAA into microdroplets reduces the assay volume to the picolitre scale, which is a ∼10^6^-fold reduction in reaction volume per polymerase assay.

This improvement in assay volume size coupled with the ability to sort >10^8^ droplets per day leads to enormous cost savings for chemically synthesized substrates like tNTPs that require more than 12 synthetic steps to produce^32^. In the case of our 8,000 member library, we performed 1 round of DrOPS using 200 μl of tNTP containing reaction buffer, which is equivalent to 20 primer-extension reactions performed under standard bulk-phase conditions. By comparison, traditional screening of the same library with 98% coverage would require 32,000 PAAs and consume >320 ml of reaction buffer. This striking difference leads to an economy of scale that benefits microfluidics-based reactions by reducing the consumption of chemically synthesized substrates, which is critical to realizing the long-term of goals of synthetic genetics^4^.

DrOPS also compares favourably with other polymerase technologies, like CSR and CST, that use w/o emulsions generated by bulk mixing^1^,^12^. While CSR and CST have been used to evolve polymerases with enhanced activity and expanded substrate recognition, both methods use the parent plasmid as template for the primer-extension reaction, which limits the range of polymerase functions to enzymes that promote DNA-templated synthesis. In addition, CST requires affinity purification on a solid-support matrix, which lowers the partitioning efficiency of functional members due to non-specific DNA binding to the resin. By contrast, DrOPS uses an optical sensor that is amenable to any nucleic acid polymer that is capable of Watson–Crick base pairing and relies on solution-based separation methods, like FACS to separate functional droplets from the non-functional pool. In addition, the ability to specify the sequence composition and length of the template provides enormous control over the stringency of the selection. Together, these properties of template control and solution-based separation make DrOPS a versatile tool that could be applied to a wide range of problems in polymerase engineering.

Although this study examined a specific problem in TNA polymerase engineering, namely, the ability to synthesize TNA in the absence of manganese ions, the DrOPS technology is unique in the sense that it could be applied to other more challenging problems in polymerase engineering. For example, the quantitative aspect of DrOPS could be used to identify new XNA polymerases with superior activity, while the template control aspect provides an avenue for discovering future polymerases that can copy XNA into DNA or possible even XNA into XNA, thereby demonstrating direct XNA replication.

In summary, we have developed a microfluidics-based method for evolving novel polymerase functions *in vitro*. The strategy functions with high partitioning efficiency, using an optical sensor that could be engineered for other substrate–template combinations. While further advances in optical detection and droplet separation are possible^36^, the ability to use commercial chips and reagents provides a technology that is readily available to most laboratories.

## Methods

### General information

DNA oligonucleotides (Supplementary Table 1) were purchased from Integrated DNA Technologies (Coralville, IA), purified by denaturing polyacrylamide gel electrophoresis, electroeluted, ethanol precipitated and quantified by ultraviolet absorbance using a NanoDrop spectrophotometer. NTPs and dNTPs were purchased from Sigma (St Louis, MO). tNTPs were obtained by chemical synthesis as previously described^1^,^2^. Accuprime DNA Polymerase was obtained from Invitrogen (Grand Island, NY). Hen egg lysozyme was purchased from Sigma. Fluorinated oil HFE-7500 was purchased from 3M Novec (St Paul, MN) and microfluidic chips were purchased from Dolomite (UK). The 9n gene was kindly provided by Andreas Marx in a pGDR11 expression vector. DNA sequencing was performed at the ASU Core Facility. Full-length gels to main text figures are provided in Supplementary Fig. 9.

### Generating emulsion droplets

The formation of w/o single emulsions was performed using a quartz glass microfluidic device with a single inlet flow-focusing junction geometry of 14 × 17 μm with a hydrophobic/fluorophilic coating (Cat. C000525G, Dolomite). The device was connected by FEP tubing through a top interface linear connector (Cat. 3000109, Dolomite) to syringes (100 μl, 500 μl SGE glass syringes, 2500 μl Hamilton Gastight syringe or 3 ml plastic syringe (Becton-Dickinson, Madrid, Spain)), which were driven by either NE1002 × syringe infusion pumps (New Era Pump Systems Inc., USA) or a pump manifold of neMESYS low pressure syringe pumps (Cetoni Gmbh, Germany) with accompanying control software. Carrier fluid was filtered using a 0.2 μm inline syringe filter, while the aqueous phase was filtered using an inline 10 μm frit filter. Droplet generation was monitored using a Nikon eclipse TS100 microscope with 20 × ELWD Nikon objective and captured using a QIclick 12 bit monochrome CCD camera (QImaging, BC Canada). Flow rates were adjusted based on visual inspection with an average rate of 5 μl min^−1^ for the aqueous phase and 12 μl min^−1^ for the carrier oil. These flow rates yielded droplets with an average diameter of 14 μm (∼1 pl volume). A low-viscosity fluorinated oil (HFE-7500) containing 1% (w/w) Pico-Surf surfactant (Dolomite) was used as the carrier fluid.

The formation of w/o/w double emulsions was performed using a quartz glass microfluidic device with a single inlet flow-focusing junction geometry of 14 × 17 μm (Cat. 3200136, Dolomite). The w/o emulsion and aqueous carrier phase were delivered to the device using syringes connected in the same manner as described above for single-emulsion formation. The w/o emulsion was slowly drawn into a 250 μl SGE glass syringe, mounted into an infusion pump in a vertical position and left to settle for at least 30 min prior to delivery. Carrier fluid (25 mM NaCl, 1% Tween-80) was filtered using a 0.2 μm inline syringe filter, while the w/o emulsion was filtered using an inline 10 μm frit filter. Flow rates were adjusted based on visual inspection with an average rate of 1 μl min^−1^ for the single emulsion and 8 μl min^−1^ for the carrier aqueous phase.

### Cell compartmentalization in droplets

Cell populations were grown and polymerase variants were expressed as described above. After expression, an aliquot (2 ml) of cell culture was centrifuged for 5 min (2,000 r.c.f.) and the supernatant discarded. The cells were washed three times with 1 × ThermoPol buffer (20 mM Tris-HCl, 10 mM (NH_4_)_2_SO_4_, 10 mM KCl, 2 mM MgSO_4_, 0.1% Triton X-100, pH 8.8). After each wash, the cells were centrifuged for 5 min (2,000 r.c.f.) and the supernatant discarded. The rinsed bacterial pellet was re-suspended in 500 μl 1 × ThermoPol buffer and the absorbance was measured at 600 nm. Cells were diluted to enable encapsulation at occupancies of 0.1 cells per droplet, according to the assumption that 1 ml of *E. coli* suspension at an *A*_600_ value of 1.0 contains 5 × 10^8^ cells. Just prior to emulsification, the cells were mixed with the fluorescence-based PAA (see section below). The w/o emulsion was collected under a layer of mineral oil in an Eppendorf tube. Following emulsification, the reactions were incubated for 5 min at 90 °C to lyse cells, followed by incubation at 55 °C for the indicated amount of time.

### Microscopy

Images were collected using a bright-field microscope (Eclipse TE300, Nikon) equipped with a Hamamatsu Orca 3CCD camera using a 60 × , 1.32 numerical aperture (NA), oil-immersion objective lens and Immersion Oil Type DF (Cargille Laboratories) imaging medium. QED InVivo 3.2 (Media Cybernetics) was used to collect images, which were processed with Photoshop CS4 (Adobe) or ImageJ (NIH) software. Microfluidic droplet generation was monitored using a Nikon eclipse TS100 inverted microscope with either a 10 × , 0.3 NA Plan fluor or 20 × , 0.45 NA ELWD S Plan Fluor, Nikon objectives and captured using a QIclick 12 bit monochrome CCD camera.

### Flow cytometric analysis of double-emulsion droplets

W/o/w double-emulsion droplets were diluted into 150 mM NaCl and subjected to flow cytometric analysis (FACSCalibur, BD Biosciences). The sample was excited with a 488 nm argon laser and the emission was detected using a 530±15 nm band-pass filter. Double-emulsion populations were gated on logFSC/logSSC. Fluorescent readout was obtained from >15,000 droplets for each measurement and analysed using Cytometer software (Cell Quest, BD Biosciences).

### Polymerase library generation

The focused 9n DNA polymerase library was generated by replacing the region coding for the finger, thumb and palm domains with a DNA cassette containing unbiased, random codons (NNN) at amino acid positions 409, 485 and 664, respectively. The DNA cassette was generated from three gBlock fragments that were combined by overlapping PCR using AccuPrime DNA polymerase (Supplementary Fig. 3). The second fragment contains a 5′ region that is conserved with the 3′ end of the first fragment and a 3′ region that is conserved with the 5′ end of the third fragment. Each fragment was individually amplified using three sets of unique primers (P1.For, P1.Rev, P2.For, P2.Rev, P3.For, P3.Rev) with an optimized number of PCR cycles determined by quantitative PCR analysis to prevent over-amplification. The full-length cassette was then assembled by combining 15 ng of each fragment and DNA primers P1.For and P3.Rev into a single PCR reaction. The PCR-amplified cassette was digested with AscI and BglII restriction enzymes, ligated into the pGDR11 expression vector and transformed into electrocompetent 10-beta *E. coli* (New England Biolabs Inc., Massachusetts, USA).

### Polymerase selections

Polymerase variants were grown as a population of *E. coli* carrying the pGDR11 plasmid encoding the polymerase of interest in Luria–Bertani (LB) broth supplemented with ampicillin (100 μg ml^−1^). Cultures were grown at 37 °C with shaking at 240 r.p.m. and protein expression was induced by adding IPTG to a final concentration of 1 mM at an OD-600 of 0.6. Induced cultures were grown for an additional 3 h at 37 °C with shaking. Prior to emulsion formation, the cells were washed three times with 1 × ThermoPol buffer (NEB, USA) and then diluted to enable encapsulation at occupancies of 0.1 cells per droplet. Just prior to emulsification, the cells were mixed with the fluorescence-based PAA. The w/o emulsion was collected under a layer of mineral oil in an Eppendorf tube. Following emulsification, the reactions were incubated for 5 min at 90 °C to lyse cells, followed by incubation at 55 °C for the indicated amount of time. Single emulsions were then converted to double emulsions as described above. Prior to sorting droplets using a FACS, the aqueous carrier phase (1% w/w Tween-80 in 25 mM NaCl) was exchanged for a solution of 25 mM NaCl to reduce the presence of surfactant in the aqueous phase. Samples were sorted in a BD FACS Aria (BD Biosciences) using PBS as a sheath fluid. A set-up with a 70 μm nozzle was chosen to give an average sort rate of 5,000–8,000 events per second. The threshold trigger was set on side scatter. The sample was excited with a 488 nm argon laser and the emission was detected using a 530±15 nm band-pass filter. The double-emulsion population was gated from other populations in the sample on logFSC/logSSC. DNA samples were recovered from sorted emulsions by extraction with ∼2 vol of Pico-Break 1 (Dolomite) to disperse the emulsions. The extracted aqueous phase was concentrated using a spin column (Zymo Research) and used to transform electrocompetent *E. coli* cells (β-10, NEB). Plasmid recovery efficiency was determined by comparing the number of sorted droplets to the number of colonies obtained after transformation and plating.

### Fluorescence-activated droplet sorting

Prior to sorting droplets using a FACS, the aqueous carrier phase (1% w/w Tween-80 in 25 mM NaCl) was exchanged for a solution of 25 mM NaCl to reduce the presence of surfactant in the aqueous phase. Samples were sorted in a BD FACS Aria using PBS as a sheath fluid. A set-up with a 70 μm nozzle was chosen to give an average sort rate of 5,000–8,000 events per second. The threshold trigger was set on side scatter. The sample was excited with a 488 nm argon laser and the emission was detected using a 530±15 nm band-pass filter. The double-emulsion population was gated from other populations in the sample on logFSC/logSSC.

### DNA recovery and transformation

DNA samples were recovered from sorted emulsions by extraction with ∼2 vol of Pico-Break 1 (Dolomite), which contains 1H,1H,2H,2H-perfluorooctanol (PFO). After addition of Pico-Break 1, the samples were vortexed, followed by centrifugation (15 s, 2,000 r.c.f.) to attain phase separation. The top, aqueous layer containing the plasmid DNA was recovered. The bottom layer was extracted second time with 1 vol of molecular grade water to improve recovery yields. The combined aqueous layers containing the plasmid DNA were concentrated using a spin column (DNA Clean & Concentrator-5, Zymo Research) and eluted with molecular biology grade water (10 μl). The DNA Clean & Concentrator-5 also facilitates removal of protein from the sample. Electrocompetent *E. coli* cells (50 μl, β-10 *E. coli* cells, NEB) were transformed with 5 μl of purified DNA by applying one electric pulse of 1.80 kV (using an *E. coli* Pulser cuvette, 0.1 cm electrode; Bio-Rad MicroPulser). Sterile S.O.C. Medium (500 μl, Invitrogen) was added immediately after pulsing and the sample was grown for 30 min at 37 °C with shaking at 240 r.p.m. before plating on LB agar containing ampicillin (100 μg ml^−1^) followed by incubation at 37 °C overnight. Plasmid recovery efficiency was determined by comparing the number of sorted droplets with the number of colonies obtained after transformation and plating. In some cases, dilution plating was used to estimate the number of successful transformants.

### Polymerase expression

Individual polymerase variants were tested by growing a clonal population of XL-1 blue *E. coli* carrying the pGDR11 plasmid encoding the polymerase of interest in LB broth supplemented with ampicillin (100 μg ml^−1^). Cultures were grown at 37 °C with shaking at 240 r.p.m. and protein expression was induced by adding IPTG to a final concentration of 1 mM at an OD-600 of 0.6. Induced cultures were grown for an additional 3 h at 37 °C with shaking. The cells were then pelleted and re-suspended in nickel-binding buffer (50 mM phosphate, 250 mM sodium chloride, 10% glycerol, pH 8) with 0.1 mg ml^−1^ hen egg lysozyme, and incubated for 1 h at 37 °C. Following lysozyme treatment, the samples were heated for 15 min at 75 °C. Aggregated cellular debris was removed by centrifugation for 15 min at 3,200 r.c.f.. Polymerases were purified from the lysate based on an N-terminal 6 × His-tag by binding to a nickel-affinity resin. After binding, the resin was washed three times with nickel-binding buffer followed by elution with nickel-binding buffer supplemented with 75 mM imidazole. Protein expression was confirmed by SDS–PAGE analysis with coomassie blue staining. Polymerases were exchanged into storage buffer (10 mM Tris-HCl, 100 mM KCl, 1 mM DTT, 0.1 mM EDTA, pH 7.4) using a Microcon-30 kDa column (Millipore, USA) and stored at 4 °C.

### Polymerase activity assays

Polymerase activity was evaluated as the ability to extend a DNA primer–template complex with natural, non-cognate and unnatural nucleotide triphosphates. Primer-extension reactions were analysed by denaturing PAGE or fluorescence. The primer–template complex was annealed in ThermoPol buffer (1 × : 20 mM Tris-HCl, 10 mM (NH4)2SO4, 10 mM KCl, 2 mM MgSO4, 0.1% Triton X-100, pH 8.8; New England Biolabs) by heating for 5 min at 95 °C and cooling for 5 min at 4 °C. Nucleotide triphosphates (100 μM final) and polymerase were added to the reaction after primer annealing and the reaction was incubated at 55 °C for the indicated amount of time. Fluorescence-based PAAs were performed using an unlabelled DNA primer, a template with a fluorophore label at the 5′ end and a quencher probe labelled with a quencher dye at the 3′ end. The concentration of primer, template and quencher strands were 2 μM, 1 μM and 3 μM, respectively. Fluorescence was measured using a 2014 EnVision multilabel plate reader (PerkinElmer). For PAGE assays, the DNA primer carried an IR800 fluorophore label at the 5′ end and an unlabelled DNA template strand. The concentration of primer and template were 0.5 μM and 1 μM, respectively, and no quencher strand was added. Reactions were quenched by adding 10 equiv. of stop buffer (1 × Tris-boric acid buffer, 20 mM EDTA, 7 M urea, pH 8). Samples were denatured for 5 min at 90 °C prior to separation by denaturing PAGE and visualization of the IR800 dye using a LICOR Oddysey CLx imager.

For the polymerase time courses, the reaction volume was increased to 25 μl. At each desired time point, 1 μl of the reaction was removed and added to 30 μl of stop buffer. Samples were then denatured for 5 min at 90 °C prior to separation by denaturing PAGE and visualization of the IR800 dye using a LICOR Oddysey CLx imager. The amount of full-length and truncated products were quantified using the Image Studio software version 4.0. All time course assays were completed with the PBS2-IR800 DNA primer and ST.1G DNA template.

### Fidelity analysis

Fidelity reactions were performed by sequencing the cDNA strand following a complete cycle of transcription and reverse transcription. The primer–template complex was extended in a 100 μl reaction volume containing 100 pmol of fidelity.temp and 100 pmol of PBS2.mismatch primer. The primer and template were annealed in 1 × ThermoPol buffer by heating for 5 min at 95 °C and cooling for 10 min at 4 °C. The 9n-YRI polymerase (10 μl) was added to the reaction mixture. For TNA extensions in the presence of Mn^2+^, the polymerase was pretreated with 1 mM MnCl_2_. The reactions were initiated by addition of the TNA nucleotide triphosphates (100 μM). Following a 4-h incubation with Mn^2+^ or an 18-h incubation without Mn^2+^ at 55 °C, the reactions were quenched in stop buffer and denatured at 90 °C for 5 min. Elongated primers were purified by denaturing PAGE, electroeluted and concentrated using a YM-30 concentrator device.

The purified transcripts were reverse transcribed in a final volume of 100 μl. PBS1 primer (100 pmol) was annealed to the template in 1 × First Strand Buffer (50 mM Tris-HCl, 75 mM KCl, 3 mM MgCl_2_, pH 8.3) by heating for 5 min at 90 °C and cooling for 10 min at 4 °C. Next, 500 μM dNTPs and 10 mM DTT were added and the reaction was allowed to incubate for 2 min at 42 °C. Finally, 3 mM MgCl_2_, 1.5 mM MnCl_2_ and 10 U μl^−1^ SuperScript II reverse transcriptase were added and the reaction was allowed to incubate for 1 h at 42 °C.

After reverse transcription, the PCR-amplified DNA (1 pmol) was ligated into a pJET vector following manufacturer's protocol. The ligated product was transformed into XL-1 blue *E. coli*, grown in liquid media and individual colonies were isolated, cloned and sequenced (ASU Core Facility). Sequencing results were analysed using CLC Main Workbench. Sequences lacking the T to A watermark were discarded as they were generated from the starting DNA template rather than replicated material. The error rate for each of the nine possible substitution (for example, T→C, T→G or T→A) was determined as follows: *μ*_exp→obs_=(#observed/#expected) × 1,000. The total error rate was determined by summing the error rate for each substitution.

## Additional information

**How to cite this article:** Larsen, A. C. *et al.* A general strategy for expanding polymerase function by droplet microfluidics. *Nat. Commun.* 7:11235 doi: 10.1038/ncomms11235 (2016).

## Supplementary Material

Supplementary Information Supplementary Figures 1-9 and Supplementary Tables 1-2
