# Supplementary material for: A general strategy for expanding polymerase function by droplet microfluidics
Source: Nat Commun. 2016 Apr 5;7:11235. doi: 10.1038/ncomms11235 (PMC4822039; doi:10.1038/ncomms11235)
Supplement: Supplementary Information — Supplementary Figures 1-9 and Supplementary Tables 1-2 [file ncomms11235-s1.pdf]

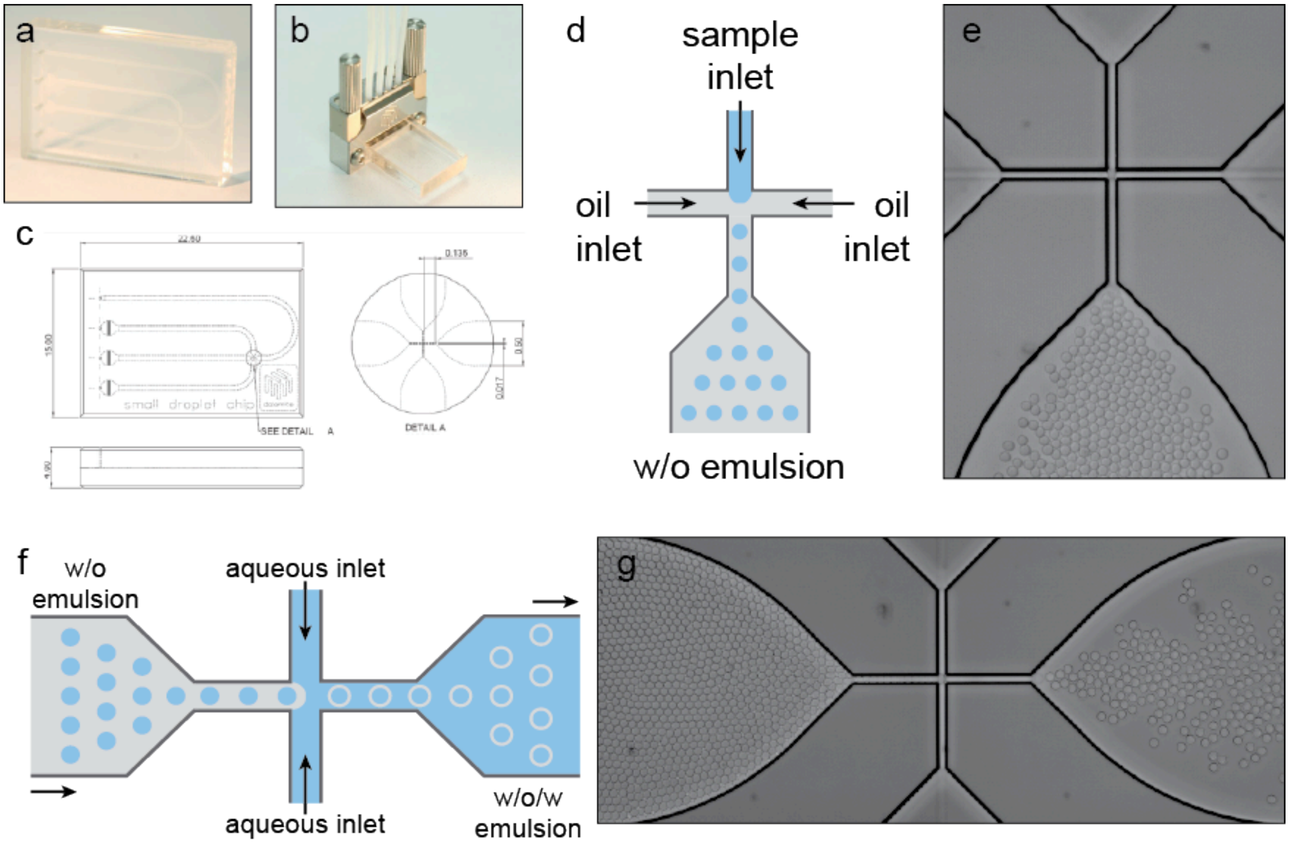

Supplementary Figure 1. Schematic and photographs of microfluidic chips and droplet formation. Photograph of microfluidic chip with, **a**, and without, **b**, the syringe interface. **c**, CAD drawing detailing chip and junction dimensions. Figures a-c provided courtesy of Dolomite Microfluidics (www.dolomite-microfluidics.com). **d**, Schematic and, **e**, photograph of the fluorophilic droplet chip producing water-in-oil emulsions. **f**, Schematic and, **g**, photograph of the hydrophilic chip that converts a water-in-oil emulsion in bulk oil phase to a water-in-oil-in-water emulsion in bulk aqueous phase.

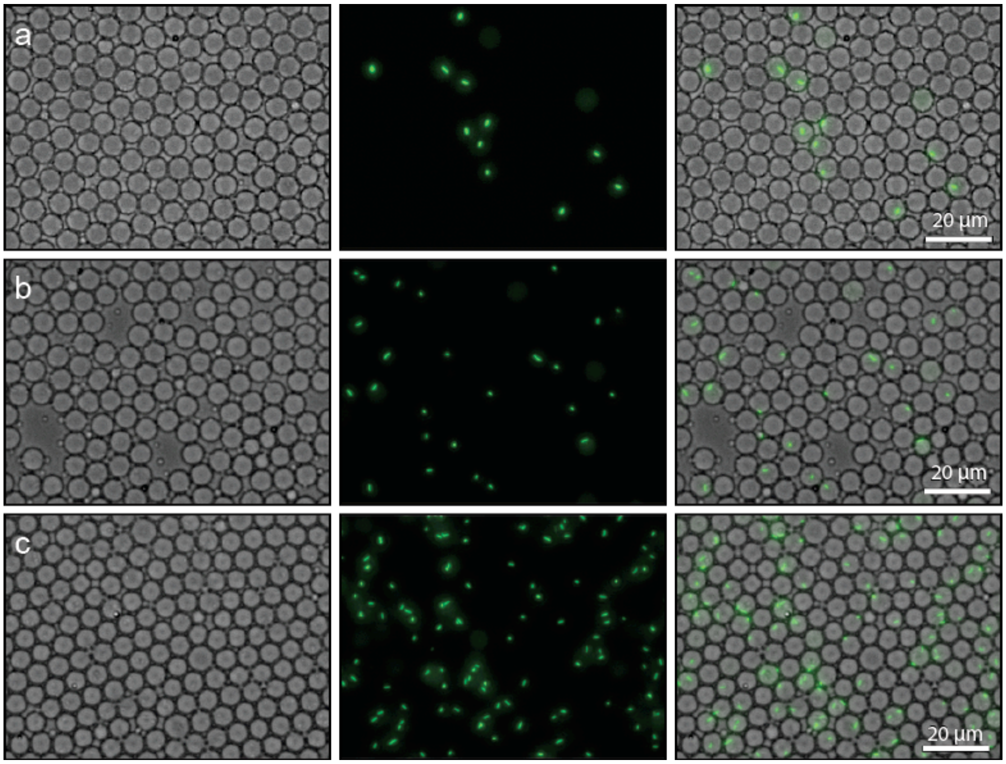

Supplementary Figure 2. *E. coli* titration to confirm droplet occupancy. GFP expressing bacteria poised at an OD-600 of **a,** 0.5, **b,** 1.0, and **c,** 2.0 were encapsulated in w/o droplets. Representative bright field, fluorescence, and overlay images of the w/o droplets are shown (left to right). Average occupancy was determined by counting the number of fluorescent bacteria per droplet.

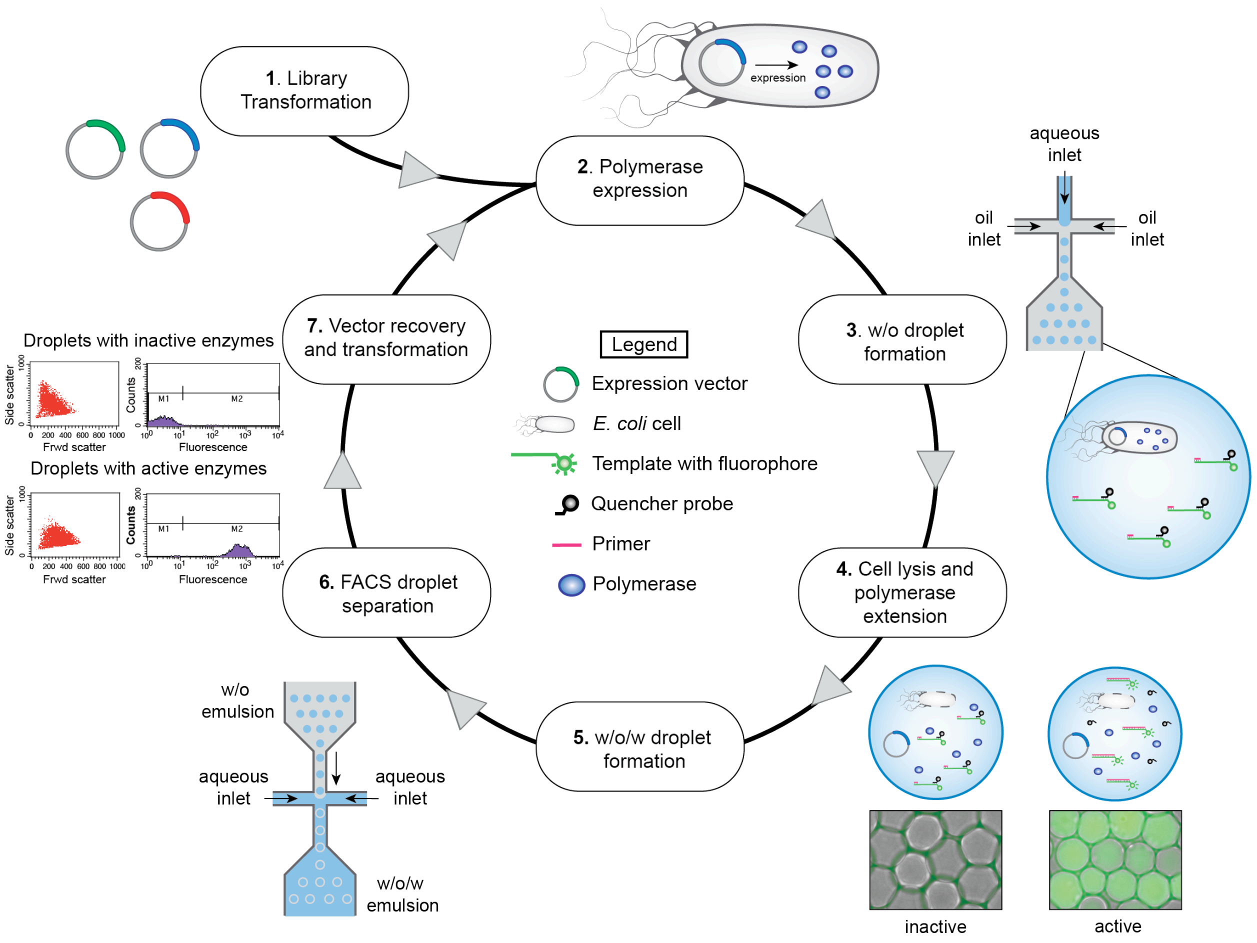

Supplementary Figure 3. Polymerase evolution strategy developed for droplet microfluidics (DrOPS). **1**, A library of DNA polymerase variants is transformed into *E. coli*. **2**, The *E. coli* cells are grown to log phase in liquid media and induced with IPTG. **3,** Water-in-oil (w/o) droplets are generated microfluidically to produce a population of artificial compartments that contain on average one *E. coli* cell per occupied compartment and a fluorescence-based polymerase activity assay. **4**, Polymerases are released from the *E. coli* by heat-induced lysis. The droplets are then incubated at 55°C during which time the polymerases are challenged to extend the primer-template complex. At this point, qualitative analysis of the population can be performed by fluorescence and bright field microscopy. **5**, The w/o droplets are then passed through a second microfluidic device to generate water-in-oil-in-water (w/o/w) emulsions in a bulk aqueous phase. **6**, Fluorescence-activated cell sorting (FACS) is used to isolate droplets that generate strong fluorescence due to the activity of a functional polymerase. **7**, Encoding plasmid DNA is recovered from sorted droplets by aqueous extraction and transformed into a new population of *E. coli* to initiate another round of selection or analyzed by DNA sequencing.

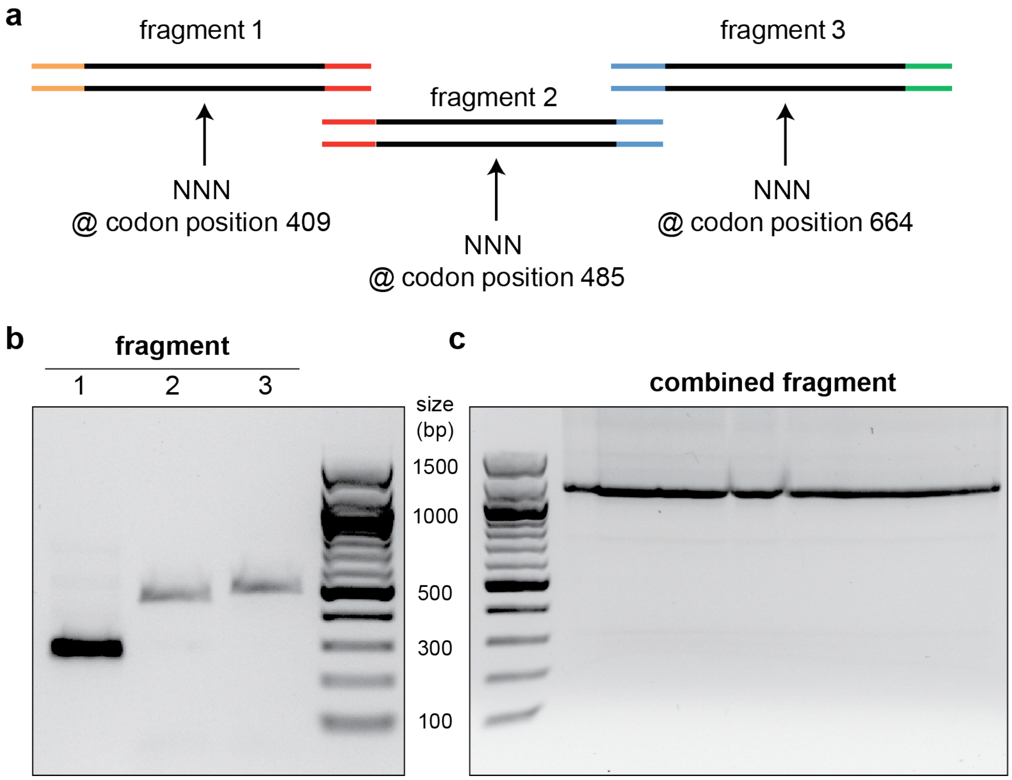

Supplementary Figure 4. Generation of a focused polymerase library. **a**, Three gBlock dsDNA fragments spanning the entire finger, palm, and thumb domains of 9n DNA polymerase were purchased from IDT with fully degenerate codons at positions 409, 485 and 664. Each fragment contained a complementary region for overlapping PCR. **b**, Each of the three fragments was individually amplified. **c**, Amplicons from each fragment were combined into a single PCR reaction using the forward primer for fragment one and the reverse primer for fragment three. The full-length fragment was combined with the remaining polymerase by restriction digestion, ligation and transformation.

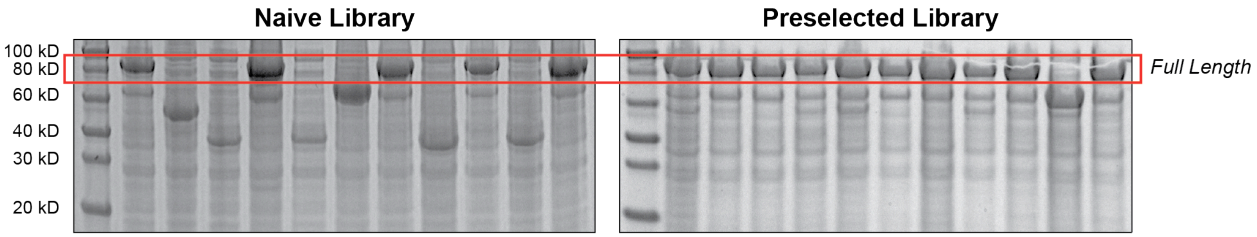

Supplementary Figure 5. Neutral drift selection for functional polymerases. One round of DrOPS was performed on the focused library in the presence of natural dNTP substrates to eliminate non-functional members. Denaturing PAGE analysis of 10 members from the naïve library (left) and the pre-selected library (right). Wild type 9n DNA polymerase was run in the first lane next to the ladder. Full-length products indicated by the red box.

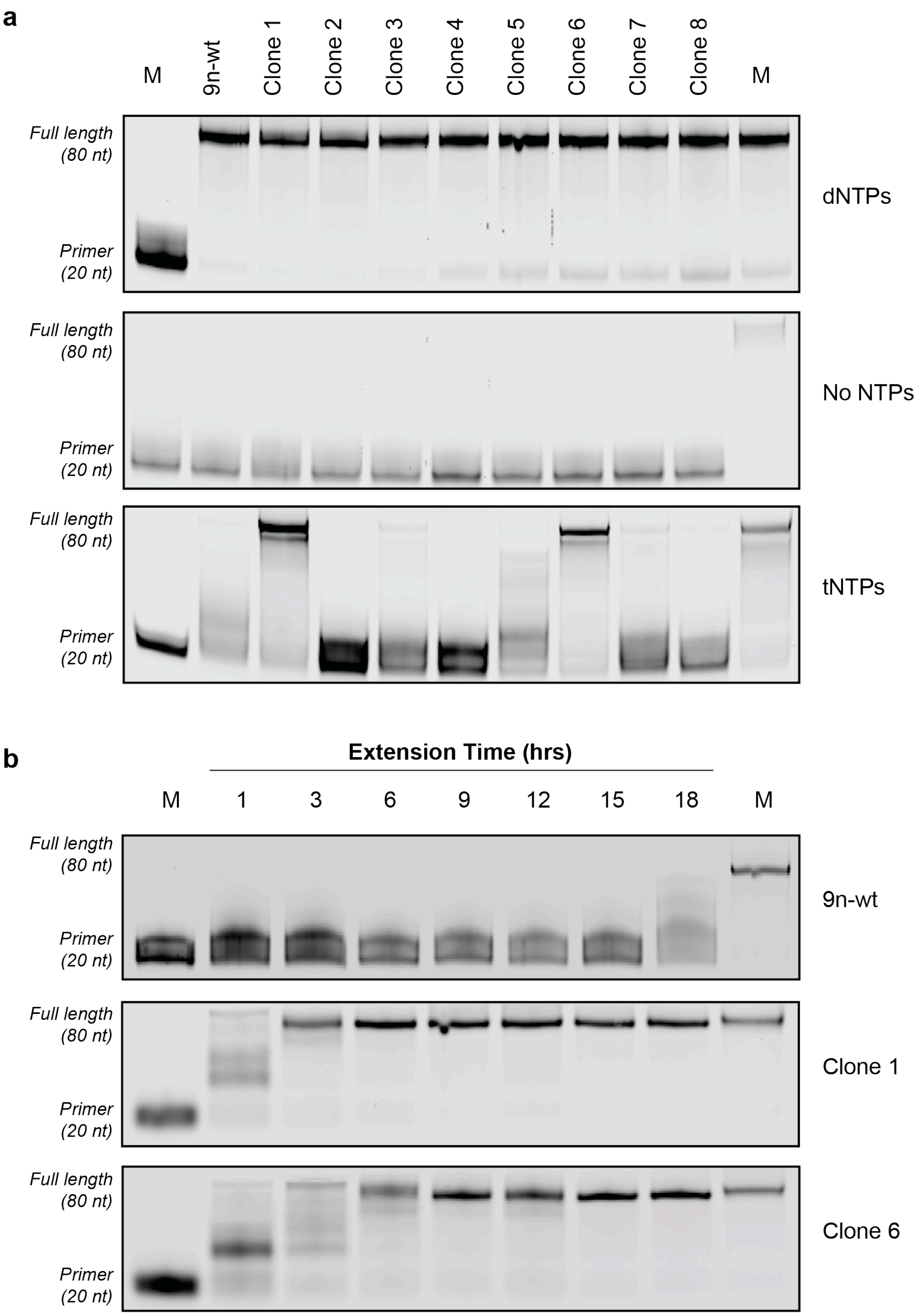

Supplementary Figure 6. Manganese-independent polymerase activity assays. All assays were completed with PBS2-IR800 DNA primer and ST.1G DNA template. Marker lanes (M) are control reactions run either in the absence of polymerase (primer only) or in the presence of dNTP substrate for 1 hour at 55ºC (full-length). **a,** Selected clones were tested in the presence of dNTP substrate for 1 hour at 55ºC and in the absence of added substrate for 18 hours at 55ºC to ensure that the recombinant polymerases were properly folded, functional, and free from cellular contaminants. Eight in vitro selected clones were tested for TNA synthesis activity by incubating with tNTPs for 18 hours at 55ºC. **b,** Time course analysis of TNA synthesis for three engineered polymerase variants in the presence of tNTP substrates.

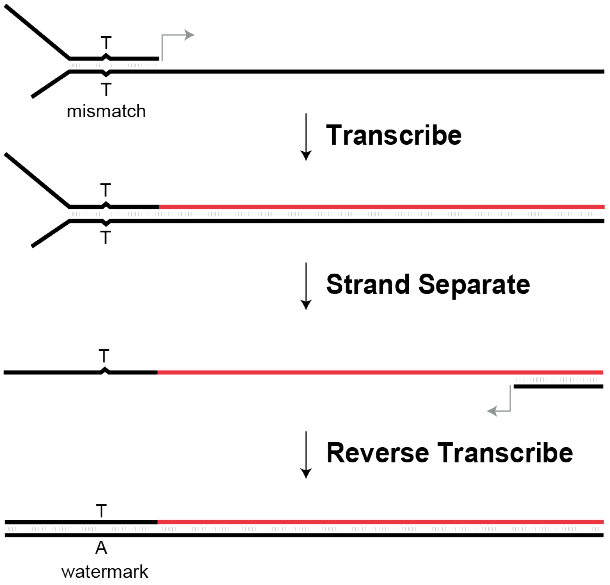

Supplementary Figure 7. Replication strategy used to measure TNA fidelity.
Schematic representation of the transcription and reverse transcription process used to evaluate the fidelity of TNA replication. DNA is shown in black, TNA is shown in red. The primer-template complex contains a T-T mismatch, which produces a T to A transversion in the cDNA strand. The transversion represents a watermark to ensure that the sequenced DNA was produced by TNA transcription and reverse transcription.

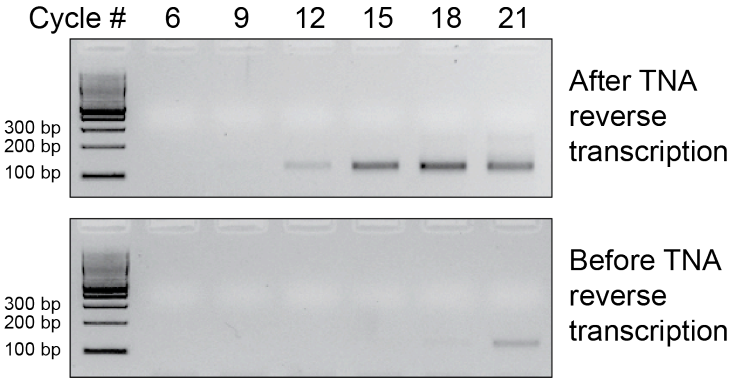

Supplementary Figure 8. Control assay for TNA purity.
PCR was used to test for DNA contamination prior to TNA reverse transcription. DNA amplification before (bottom) and after (top) reverse transcription of the TNA product into cDNA. cDNA amplifies approximately 9 cycles earlier than the contaminating DNA template, representing an ~500-fold excess over background or 99% pure.

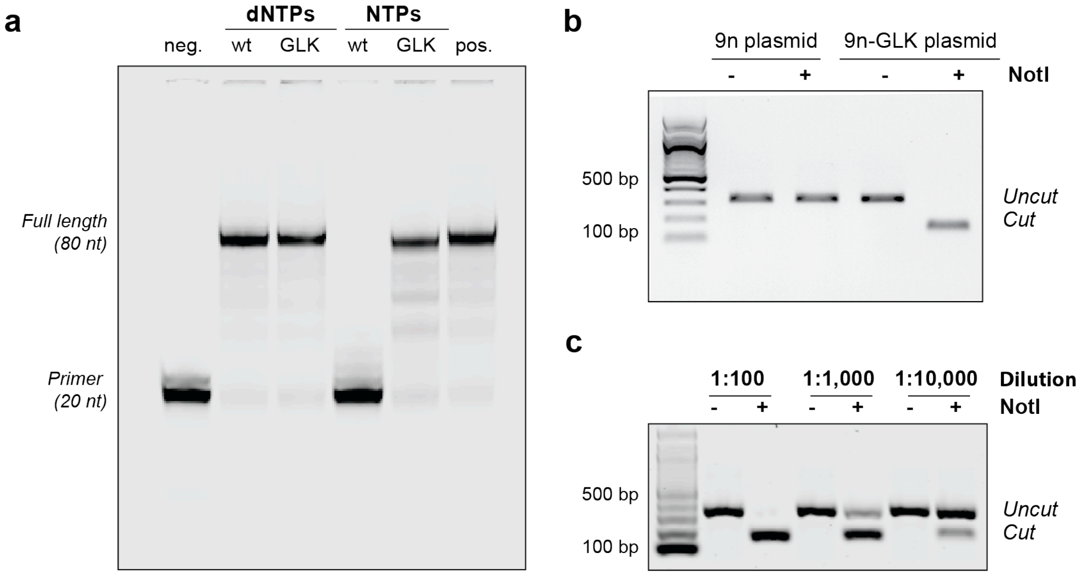

Supplementary Figure 9. Full gel images for main text figures.
The full gels are provided for main text Figure 1d, 2b, and 2c.

Supplementary Table 1. DNA primer and template sequences. Modifications are written following IDT nomenclature. N represents a degenerate position containing an equal distribution of A, T, G, and C nucleobases. Engineered primer mismatches are denoted in lowercase.

Name	DNA Sequence (5' -> 3')
Lib.409.NNN	GAACGTGAACTGGCGCGCCGTCGTGGCGGTTATGCGGGCGGTTATGTGAAAGAA<br>CCGGAACGTGGCCTGTGGGATAACATTGTGTATCTGGATTTTCGTAGCCTG**NNN**<br>CCGAGCATTATTATCACCCACAATGTGAGCCCGGATACCCTGAACCGTGAAGGC<br>TGCAAAGAATATGATGTGGCGCCGGAAGTGGGCCATAAATTCTGCAAAGATTTC<br>CCGGGCTTTATT
Lib.485.NNN	AAGATTTCCCGGGCTTTATTCCGAGCCTGCTGGGCGATCTGCTCGAGGAACGCC<br>AGAAAATCAAACGCAAAATGAAAGCGACCGTTGATCCGCTGGAAAAAAAACTGC<br>TGGATTATCGTCAGCGC**NNN**ATTAAAATTCTGGCCAACAGCTTCTATGGCTATT<br>ATGGTTATGCGAAAGCGCGTTGGTATTGCAAAGAATGCGCGGAAAGCGTGACCG<br>CGTGGGGCCGTGAATATATCGAAATGGTGATCCGCGAGCTCGAAGAAAAATTCG<br>GCTTCAAAGTGCTGTATGCGGATACCGATGGCCTGCATGCGACCATTCCGGGTG<br>CGGATGCGGAAACCGTGAAAAAAAAAGCGAAAGAATTCCTGAAATACATCAATC<br>CGAAACTGCCGGGCCTGCTGGAACTGGAATATGAAGGCTTTTATGTGCGTGGCT<br>TTTTCGTGACCAAAAAAAAATACGCGGTGATCGATGAAGAAGGCAAAATTACCA<br>CCCGTGGCCTGGAA
Lib.664.NNN	AATACGCGGTGATCGATGAAGAAGGCAAAATTACCACCCGTGGCCTGGAAATTG<br>TGCGTCGTGATTGGAGCGAAATTGCGAAAGAAACCCAGGCGCGTGTGCTGGAAG<br>CGATTCTGAAACATGGCGATGTGGAAGAAGCGGTGCGTATTGTTAAAGAAGTGA<br>CCGAAAAACTGAGCAAATATGAGGTACCGCCGGAAAAACTGGTGATTCAT**NNN**C<br>AAATTACCCGTGATCTGCGTGATTATAAAGCGACCGGTCCGCATGTGGCGGTGG<br>CAAAACGTCTGGCAGCGCGTGGCGTGAAAATTCGTCCGGGCACCGTGATTAGCT<br>ATATTGTGCTGAAAGGCAGCGGCCGCATTGGCGATCGTGCGATTCCGGCGGATG<br>AATTTGATCCGACCAAACATCGTTATGATGCGGAATATTATATCGAAAACCAGG<br>TGCTGCCGGCGGTGGAACGTATTCTGAAAGCGTTTGGCTATCGTAAAGAAGATC<br>TGCGCTATC
P1.For	AACTGGCGCGCCGTCGTGGCGGTTATGCGG
P1.Rev	CGTTCCTCGAGCAGATCGCCCAGCAGGCTCGGAATAAAG
P2.For	ATCTGCTCGAGGAACGCCAGAAAATCAAACGC
P2.Rev	TTCCAGGCCACGGGTGGTAATTTTGC
P3.For	AATACGCGGTGATCGATGAAG
P3.Rev	GATAGCGCAGATCTTCTTTACGATAGCC
PBS2-IR800	/5IRD800/GACACTCGTATGCAGTAGCC
ST.1G.Cy3	/5Cy3/ACAACCATACTCTCCTCATCACTATTCAACTTACAATCGATACAACCT<br>TATAATCCACATGGCTACTGCATACGAGTGTC
ST.1G.FAM	/56FAM/ACAACCATACTCTCCTCATCACTATTCAACTTACAATCGATACAACC<br>TTATAATCCACATGGCTACTGCATACGAGTGTC
ST.1G	ACAACCATACTCTCCTCATCACTATTCAACTTACAATCGATACAACCTTATAAT<br>CCACATGGCTACTGCATACGAGTGTC
QP13.Iowa	AGAGTATGGTTGT/3IABkFQ/
QP16.Iowa	AGGAGAGTATGGTTGT/3IABkFQ/
QP20.Iowa	GATGAGGAGAGTATGGTTGT/3IABkFQ/

QP13.BHQ	AGAGTATGGTTGT/3BHQ_1/
QP16.BHQ	AGGAGAGTATGGTTGT/3BHQ_1/
QP20.BHQ	GATGAGGAGAGTATGGTTGT/3BHQ_1/
Fidelity.Temp	TGTCTACACGCAAGCTTACATTAAGACTCGCCATGTTACGATCTGCCAAGTACA GCCTTGAATCGTCACTGGCTACTGCATACGAGTGTC /3InvdT/
PBS2.Mismatch	CTTTTAAGAACCGGACGAACGACACTCGTtTGCAGTAGCC
PBS1	TGTCTACACGCAAGCTTACA
Extra.Primer	CTTTTAAGAACCGGACGAAC

Supplementary Table 2. DNA sequencing results from aggregate fidelity assay performed in the absence of Mn^{2+}. PBS2.mismatch and PBS1 primer sites are underlined. Control watermark (shown in red) confirmed the cDNA read underwent TNA replication (see Figure SI 7).

Read 1	CTTTTAAGAACCGGACGAACGACACTCGT**T**TGCAGTAGCCCCATTCTTTAACAGC<br>TCATCACTAGACATTTATAAGTCAACATTAACCTCTGTCTACACGCAAGCTTACA
Read 2	CTTTTAAGAACCGGACGAACGACACTCGT**T**TGCAGTAGCCCCATTCTTTAACAGC<br>TCATCACTAGACATTTATAAGTCAACATTAACCTCTGTCTACACGCAAGCTTACA
Read 3	CTTTTAAGAACCGGACGAACGACACTCGT**T**TGCAGTAGCCCCATTCTTTAACAGC<br>TCATCACTAGACATTTATAAGTCAACATTAACCTCTGTCTACACGCAAGCTTACA
Read 4	CTTTTAAGAACCGGACGAACGACACTCGT**T**TGCAGTAGCCCCATTCTTTAACAGC<br>TCATCACTAGACATTTATAAGTCAACATTAACCTCTGTCTACACGCAAGCTTACA
Read 5	CTTTTAAGAACCGGACGAACGACACTCGT**T**TGCAGTAGCCCCATTCTTTAACAGC<br>TCATCACTAGACATTTATAAGTCAACATTAACCTCTGTCTACACGCAAGCTTACA
Read 6	CTTTTAAGAACCGGACGAACGACACTCGT**T**TGCAGTAGCCCCATTCTTTAACAGC<br>TCATCACTAGACATTTATAAGTCAACATTAACCTCTGTCTACACGCAAGCTTACA
Read 7	CTTTTAAGAACCGGACGAACGACACTCGT**T**TGCAGTAGCCCCATTCTTTAACAGC<br>TCATCACTAGACATTTATAAGTCAACATTAACCTCTGTCTACACGCAAGCTTACA
Read 8	CTTTTAAGAACCGGACGAACGACACTCGT**T**TGCAGTAGCCCCATTCTTTAACAGC<br>TCATCACTAGACATTTATAAGTCAACATTAACCTCTGTCTACACGCAAGCTTACA
Read 9	CTTTTAAGAACCGGACGAACGACACTCGT**T**TGCAGTAGCCCCATTCTTTAACAGC<br>TCATCACTAGACATTTATAAGTCAACATTAACCTCTGTCTACACGCAAGCTTACA
Read 10	CTTTTAAGAACCGGACGAACGACACTCGT**T**TGCAGTAGCCCCATTCTTTAACAGC<br>TCATCACTAGACATTTATAAGTCAACATTAACCTCTGTCTACACGCAAGCTTACA
Read 11	CTTTTAAGAACCGGACGAACGACACTCGT**T**TGCAGTAGCCCCATTCTTCAACAGC<br>TCATCACTAGACATTTATAAGTCAACATTAACCTCTGTCTACACGCAAGCTTACA
Read 12	CTTTTAAGAACCGGACGAACGACACTCGT**T**TGCAGTAGCCCCATTCTTTAACAGC<br>TCATCACTAGACATTTATAAGTCAACATTAACCTCTGTCTACACGCAAGCTTACA
Read 13	CTTTTAAGAACCGGACGAACGACACTCGT**T**TGCAGTAGCCCCATTCTTTAACAGC<br>TCATCACTAGACATTTATAAGTCAACATTAACCTCTGTCTACACGCAAGCTTACA
Read 14	CTTTTAAGAACCGGACGAACGACACTCGT**T**TGCAGTAGCCCCATTCTTTAACAGC<br>TCATCACTAGACATTTATAAGTCAACATTAATCTCTGTCTACACGCAAGCTTACA
Read 15	CTTTTAAGAACCGGACGAACGACACTCGT**T**TGCAGTAGCCCCATTCTTTAACAGC<br>TCATCACTAGACATTTATAAGTCAACATTAACCTCTGTCTACACGCAAGCTTACA
Read 16	CTTTTAAGAACCGGACGAACGACACTCGT**T**TGCAGTAGCCCCATTCTTTAACAGC<br>TCATCACTAGACATTTACAAGTCAACATTAACCTCTGTCTACACGCAAGCTTACA
Read 17	CTTTTAAGAACCGGACGAACGACACTCGT**T**TGCAGTAGCCCCATTCTTTAACAGC<br>TCATCACTAGACATTTATAAGTCAACATTAACCTCTGTCTACACGCAAGCTTACA
Read 18	CTTTTAAGAACCGGACGAACGACACTCGT**T**TGCAGTAGCCCCATTCTTTAACAGC<br>TCATCACTAGACATTTATAAGTCAACATTAACCTCTGTCTACACGCAAGCTTACA
Read 19	CTTTTAAGAACCGGACGAACGACACTCGT**T**TGCAGTAGCCCCATTCTTTAACAGC<br>TCATCACTAGACATTTATAAGTCAACATTAACCTCTGTCTACACGCAAGCTTACA
Read 20	CTTTTAAGAACCGGACGAACGACACTCGT**T**TGCAGTAGCCCCATTCTTTAACAGC<br>TCATCACTAGACATTTATAAGTCAACATTAACCTCTGTCTACACGCAAGCTTACA
Read 21	CTTTTAAGAACCGGACGAACGACACTCGT**T**TGCAGTAGCCCCATTCTTTAACAGC<br>TCATCACTAGACATTTATAAGTCAACATTAACCTCTGTCTACACGCAAGCTTACA
Read 22	CTTTTAAGAACCGGACGAACGACACTCGT**T**TGCAGTAGCCCCATTCTTTAACAGC<br>TCATCACTAGACATTTATAAGTCAACATTAACCCCTGTCTACACGCAAGCTTACA
Read 23	CTTTTAAGAACCGGACGAACGACACTCGT**T**TGCAGTAGCCCCATTCTTTAACAGC<br>TCATCACTAGACATTTATAAGTCAACATTAACCTCTGTCTACACGCAAGCTTACA

Read 24	CTTTTAAGAACCGGACGAACGACACTCGT**T**TGCAGTAGCCCCATTCTTTAACAGC
	TCATCACTAGACATTTATAAGTCAACATTAACCTCTGTCTACACGCAAGCTTACA
Read 25	CTTTTAAGAACCGGACGAACGACACTCGT**T**TGCAGTAGCCCCATTCTTTAACAGC
	TCATCACTAGACATTTATAAGTCAACAATAACCTCTGTCTACACGCAAGCTTACA
